# Chronic spinal cord injury is associated with morphometric brain changes in functional networks beyond the sensorimotor system

**DOI:** 10.3389/fneur.2025.1672506

**Published:** 2025-12-08

**Authors:** Lorenzo Diana, Jothini Sritharan, Vanessa Vallesi, Nicola Brunello, Rajeev K. Verma, Giuseppe A. Zito

**Affiliations:** 1 Swiss Paraplegic Research, Nottwil, Switzerland; 2 Faculty of Health Sciences and Medicine, University of Lucerne, Lucerne, Switzerland; 3 Support Centre for Advanced Neuroimaging (SCAN), Institute for Diagnostic and Interventional Neuroradiology, Inselspital, Bern University Hospital and University of Bern, Bern, Switzerland; 4 ARTORG Center for Biomedical Engineering Research, University of Bern, Bern, Switzerland; 5 Swiss Paraplegic Centre, Nottwil, Switzerland

**Keywords:** chronic spinal cord injury, secondary health issues, voxel-based morphometry, surface-based morphometry, functional networks

## Abstract

**Introduction:**

Spinal cord injury (SCI) leads to motor and sensory deficits, triggering widespread neurodegeneration across the central nervous system. While sensorimotor brain changes have been widely studied, growing evidence suggests that SCI also affects regions involved in cognition, emotion, and pain regulation—domains frequently altered in the chronic stage. However, morphological alterations in higher-order brain networks remain poorly characterized. To address this gap, we investigated structural brain changes in chronic SCI using voxel-based and surface-based morphometry, focusing on large-scale functional networks.

**Methods:**

We retrospectively analyzed high-resolution T1-weighted MRI data from 45 individuals with chronic traumatic SCI and 45 matched controls. Gray matter volume and cortical thickness were estimated with CAT12. Group comparisons and associations with clinical measures—including time since injury, sensorimotor impairment, and chronic pain—were assessed using whole-brain and region-based morphometry mapped to the Schaefer functional atlas.

**Results:**

Compared to controls, SCI participants showed reduced volume in the precentral gyrus (sensorimotor network), increased volume in the right precuneus (executive control network) and reduced cortical thickness in the left temporal pole (limbic network). Longer time since injury and greater sensorimotor deficits were associated with atrophy in sensorimotor, temporo-parietal, and prefrontal regions spanning executive, attentional, default mode, and salience networks. Additionally, chronic pain was linked to atrophy in the sensorimotor cortex, basal ganglia, and cerebellum.

**Discussion:**

These findings suggest that chronic SCI is associated with widespread morphometric changes beyond the sensorimotor system. Such alterations may underlie cognitive, emotional, and pain-related symptoms, and represent potential biomarkers of secondary health complications following SCI.

## 1 Introduction

Spinal cord injury (SCI) can lead to partial or complete loss of motor, sensory, and autonomic functions below the level of injury, with profound consequences on physical and social well-being (1, 2). Following the initial cascade of pathophysiological events at the injury site, degenerative changes can propagate throughout the central nervous system (CNS), leading to gray matter (GM) and white matter atrophy affecting both the brain and spinal cord (3). These changes are detectable within the first few months post-injury (4, 5), and further progress into the chronic stage (6), i.e., after 1 year post-injury (7).

Magnetic resonance imaging (MRI) plays a central role in detecting micro- and macrostructural changes in the CNS following SCI, e.g., changes in volume, myelin integrity, and iron content (8), and quantitative imaging techniques are establishing as gold-standard biomarkers for the prediction of short- and long-term neurological and functional recovery (6, 9–11). A consistent body of MRI research has shown that structural reorganization of the CNS following SCI mostly occurs in cortical and subcortical sensorimotor circuits, including the primary and secondary sensorimotor areas (4–6, 10–12), the cortico-spinal tracts (4, 13), the brainstem (14), the thalamus and the basal ganglia (6, 14, 15), as well as the cerebellum (6, 16). Nonetheless, remote changes in the brain following SCI are not limited to the main sensorimotor pathways, but also extend to regions involved, for instance, in emotional and pain processing, as well as cognitive functioning (e.g., the orbitofrontal cortex, the superior temporal gyrus, as well as the superior and medial frontal gyrus) (3, 17–20). Such changes may become more evident in the chronic stage of SCI, where emotional distress and sustained neuropathic pain may affect the structural and functional reorganization of the brain (21, 22). For instance, the presence of neuropathic pain, a chronic condition that affects most of the SCI individuals (23), is commonly associated with structural changes within the limbic network, such as the insula and the cingulate cortex (14, 21, 24).

Studies on functional connectivity have further corroborated the importance of characterizing brain changes in higher order networks, especially in chronic SCI. For instance, studies based on resting-state functional connectivity (rsFC) have demonstrated complex patterns of altered rsFC between sensorimotor networks and networks associated with high-order functions, such as the ventral and dorsal attentional networks (VAN and DAN), as well as the executive control network (ECN) and the default mode network (DMN) (5, 25–28). These networks play an important role for processes such as attention, executive functioning, emotion regulation and pain processing (29–31), and their investigation could help identify neural biomarkers of secondary health conditions that develop and worsen over the chronic stage of disease, including cognitive impairments (32).

However, despite the growing body of rsFC studies, knowledge about structural brain alterations in chronic SCI beyond the primary sensorimotor circuits is limited and non-conclusive, often based on small sample sizes and sub-group analyses. Given the importance of the above-mentioned functional networks for several higher-order processes that may be affected in chronic SCI, investigating the structural reorganization of such systems could shed light on the broader neurobiological consequences of SCI.

The present retrospective study addresses this gap by (1) investigating morphological brain changes focusing on individuals with chronic SCI, and (2) by adopting a functionally-informed, network-based perspective (33)—a framework that has not yet been systematically applied to brain morphometry in SCI—to ease the comprehension structural, functional, and behavioral alterations observed in this population.

Specifically, besides conventional whole-brain analyses, we examined GM volume and cortical thickness in regions defined by their contribution to known resting-state functional networks. As primary outcome, we assessed differences between individuals with chronic SCI and a group of non-injured participants (non-SCI). As secondary outcomes, we explored the effects of time since injury (TSI) and the severity of sensorimotor impairments on structural brain reorganization. Finally, we explored differences in brain morphometry related to chronic pain. We considered both changes GM volume and cortical thickness, as they capture complementary aspects of cortical structure. In particular, GM volume reflects a combination of thickness, surface area, and gyrification, whereas cortical thickness specifically measures the distance between the white matter and pial surfaces, reflecting cortical laminar architecture (34). Both metrics have been employed in previous studies, and have proven useful for characterizing morphological brain changes following SCI (6, 16).

Our primary hypothesis was that chronic SCI is associated to widespread morphometric alterations beyond the sensorimotor system, as compared to a group of non-injured controls. Secondarily, at an exploratory level, we hypothesized that these changes become more pronounced over time and with greater clinical impairment. By integrating structural imaging within a network-based perspective, this study aims to provide a more comprehensive understanding of supraspinal reorganization in chronic SCI beyond the sensorimotor systems.

## 2 Materials and methods

### 2.1 Participants

In this retrospective, cross-sectional study, we analyzed data from 45 individuals with chronic SCI (mean age ± SD = 44.4 ± 11.1 years; 13F/32M; mean TSI = 13.2 ± 10.4 years) and 45 individuals without SCI (non-SCI; mean age = 40.8 ± 12.1 years; 21F/24M). The sample size was estimated with G*Power 3.1 (35), for an analysis of covariance (ANCOVA) testing between-group differences (SCI and non-SCI) in brain morphometry, with a medium effect size (*f* = 0.3), no. of covariates = 2 [total intracranial volume (TIV) and age], numerator df = 1, *α* = 0.05, and 1 − *β* = 0.8. All participants had been recruited at the Swiss Paraplegic Centre and Swiss Paraplegic Research in Nottwil, Switzerland within previous research studies; Non-SCI participants were selected to match the SCI group’s age and sex distribution as closely as possible (see Table 1; individual clinical data for the SCI group are provided in Supplementary Table S1).

**Table 1:** Demographic and clinical characteristics of participants with and without spinal cord injury (SCI).

Variable	SCI	Non-SCI	Comparison
Age (years)	44.4 ± 11.1 (19–59)	40.8 ± 12.1 (19–59)	*p* = 0.151
Sex (F/M)	13 F/32 M	21F/24M	*p* = 0.082
TSI (years)	13.2 ± 10.4 (1–40)	–	–
AIS	16 A/4 B/5 C/20 D	–	–
ISNCSCI Motor—total	72.6 ± 20.8 (37–100)	–	–
ISNCSCI Light touch Motor—total	70.2 ± 22.1 (19–110)	–	–
ISNCSCI Pinprick—Motor—total	67.6 ± 25 (10–112)	–	–
Pain	15 NP/19 nNP/11 noP		

For continuous variables, mean ± standard deviation and range are reported. AIS, American Spinal Injury Association Impairment Scale; ISNCSCI, International Standards for Neurological Classification of Spinal Cord Injury; NP, neuropathic pain; nNP, non-neuropathic pain; noP, no pain; TSI, Time Since Injury.

Inclusion criteria for the SCI group were: (i) chronic, traumatic SCI [TSI > 12 months (7)]; (ii) no history of traumatic brain injury, verified through clinical history and assessment of Fluid-Attenuated Inversion Recovery (FLAIR) MRI sequences (part of the experimental protocol), performed by a trained radiologist (R. K. V.). Further inclusion criteria, both for SCI and non-SCI, were: age ≥18 years, no history of major neurological (other than SCI) or psychiatric disorders, and no contraindications to MRI. Written informed consent was obtained from all participants. The study was approved by the Ethics Committee of Northwest and Central Switzerland (EKNZ; ID 2025–00804) and was conducted according to the Declaration of Helsinki.

### 2.2 Clinical assessment

Participants with SCI underwent a sensorimotor evaluation based on the International Standards for Neurological Classification of Spinal Cord Injury (ISNCSCI) (36). Briefly, the strength of key muscles in the upper and lower extremities was assessed using a 0–5 scale (0 = no voluntary control; 5 = full strength). Sensory integrity was evaluated through light touch and pinprick perception across all dermatomes, scored on a 0–2 scale (0 = no sensation; 1 = abnormal sensation; 2 = normal sensation). For the analyses, we considered the total motor score (range: 0–100), the total light touch score (range: 0–112), and the total pinprick score (range: 0–112). In addition, the presence of nociceptive and neuropathic pain was extracted from the clinical records.

### 2.3 Imaging acquisition and processing

All participants underwent high-resolution T1-weighted 3D imaging of the whole brain using the following parameters: field of view (FoV) = 256 × 256 × 180 mm^3^, repetition time (TR) = 8.14 ms, echo time (TE) = 3.73 ms, flip angle = 8°, slice thickness = 1 mm, and resolution = 1 × 1 × 1 mm^3^. Images were acquired using a Philips Achieva 3T MRI scanner (release: 5.4.1; Philips Healthcare, Best, Netherlands) equipped with a 32-channel head coil.

MRI data were processed using the CAT12 toolbox (v.12.9; Computational Anatomy Toolbox) (37) implemented in SPM12 (University College London, UK) within MATLAB (2019b; The MathWorks Inc., USA). The default CAT12 processing pipeline was applied to compute GM volume and surface-based cortical thickness. Briefly, individual anatomical scans were denoised and resampled before segmentation into GM, white matter, and cerebrospinal fluid (CSF) using tissue probability maps. Partial volume correction was performed, followed by normalization to standard Montreal Neurological Institute space (MNI) via Geodesic Shooting registration methods (38). Surface-based processing included cortical thickness estimation and central surface reconstruction using projection-based methods. After topological correction and surface refinement, individual surfaces were mapped to the Freesurfer “FsAverage” template (Athinoula A. Martinos Center for Biomedical Imaging, Charlestown, Massachusetts, USA). GM volume and cortical thickness maps were spatially smoothed with Gaussian kernels to reduce individual variance; full width at half maximum filters of 8 mm and 12 mm were applied for volume and cortical thickness analyses, respectively, according to https://neuro-jena.github.io/cat12-help/#intro.

To investigate morphometric changes from a network-based perspective, region-based morphometry (RBM) was employed to analyze local GM volume and cortical thickness within regions defined by the local–global intrinsic functional connectivity parcellation atlas (33, 39). This atlas delineates 100 cortical regions (50 per hemisphere) across different functional networks based on data from 1,489 subjects. For each region of the atlas, we extracted GM volume and cortical thickness.

### 2.4 Analyses

Statistical analyses were run in MATLAB and jamovi 2.4.14 (40). Data visualization was created with the functional connectivity toolbox (CONN v22.v2407) in SPM12 and RStudio (41, 42).

Between-group differences in age and sex distribution were tested with the Mann–Whitney test and the *χ*^2^ test, respectively. Associations between clinical variables (i.e., TSI and the ISNCSCI sensory and motor scores) were tested with Spearman correlations. To further characterize the clinical population, we report differences in sensory and motor scores between the left and the right sides of the body, assessed with the Wilcoxon test.

Different statistical models were then computed to identify: (i) differences in GM morphometry between SCI and non-SCI (primary outcome), (ii) associations between GM morphometry and clinical variables (including TSI and the severity of sensory and motor impairment secondary outcomes), and (iii) potential network-based morphological changes associated with the presence of neuropathic pain (secondary outcome). Both conventional whole-brain analyses and RBM analyses (i.e., the network-level investigation) were carried out. For the exploratory analyses of pain-related effects on brain morphology, the SCI cohort was divided into three subgroups based on clinical records: individuals with neuropathic pain (NP; *n* = 15), those with nociceptive but no neuropathic pain (nNP; *n* = 19), and those without pain (noP; *n* = 11). Group differences in sensorimotor impairment (ISNCSCI scores) and TSI were first assessed using Kruskal–Wallis tests.

#### 2.4.1 Differences in GM morphometry between SCI and non-SCI—primary outcome

*Whole-brain analysis.* Two-sample t-tests were conducted in SPM12 to compare morphometric variables—GM volume and cortical thickness—between SCI and non-SCI groups. For volume-based analyses, age (mean-centered), sex and TIV were included as covariates. For cortical thickness analyses, only age was included as a covariate, since brain volume is typically uncorrelated with surface-based metrics (43). Separate t-contrasts were tested in both directions: “SCI > non-SCI” (e.g., increased GM volume in SCI compared to non-SCI) and “SCI < non-SCI” (e.g., decreased GM volume in SCI compared to non-SCI). Significant peaks were identified at *p* < 0.001, uncorrected. At the cluster-level, False Discovery Rate (FDR) correction (*p_FDR_* < 0.05) was applied to account for multiple comparisons. Cluster-size thresholds were empirically defined based on voxel counts computed during model estimation (44).

*Region-based morphometry.* To examine mean differences within the functional networks defined by Schaefer’s atlas, GM volume and cortical thickness values were extracted for each region of interest of the atlas. Separate general lineal models (GLMs) were conducted with jamovi, with each brain area as the dependent variable and Group as factor. All models were covaried by Age. TIV and sex were additionally added as covariates for volume analyses. As for the whole-brain analyses, different series of analyses were run for the right and the left hemisphere. Due the high number of models tested, *α* was corrected with Bonferroni, i.e., statistical significance was set at *p* ≤ 0.001 (0.05/50 regions of the Schaefer atlas within each hemisphere).

#### 2.4.2 Associations between GM morphometry and clinical variables—secondary outcomes

*Whole-brain analysis.* To test the association between brain morphometric properties and clinical variables (i.e., motor, light touch, and pinprick scores of the ISNCSCI), we ran separate multiple regression models in SPM12, considering morphometric data of the SCI group only. As for the previous analyses, age, sex, and TIV (the latter two variables only for volume-based analyses) were entered as covariates. Negative and positive associations were tested separately, applying the same corrections for multiple comparisons as above.

*Region-based morphometry.* In the SCI sample, associations with clinical-demographic variables were tested with GLMs for volume-based analyses (covariates: age, sex, and TIV) and with Spearman correlations for cortical thickness analyses (covariate: age). In line with the whole brain analyses, positive and negative associations were tested separately. Separate sets of analyses were run for the right and the left hemisphere. Bonferroni correction was applied, and statistical significance was set at *p* ≤ 0.001.

Finally, for all brain regions that showed significant associations with clinical variables, we tested for interactions between TSI and sensorimotor impairment in predicting volume and cortical thickness. This analysis aimed to explore whether different patterns of brain reorganization emerge as a function of the interaction between injury duration and neurological severity. Given the widespread impact of motor impairment on brain morphology (see Section 3.2.2), and the observed correlations between ISNCSCI motor and sensory scores (see Section 3), motor scores were selected as the primary measure of neurological impairment. GLMs were performed with GM volume or cortical thickness in each region of interest as the dependent variable. Age, sex, TIV (the latter two variables only for volume analyses), TSI, and ISNCSCI motor scores were included as covariates, with specific focus on the interaction term between TSI and motor scores. For these models, the significance threshold was set at *α* = 0.05.

#### 2.4.3 The effect of pain on GM morphometry—secondary outcomes

*Whole-brain analysis.* In SPM12, we tested between-group differences in GM volume and cortical thickness with a full factorial design. Age, sex and TIV (the latter two variables only for volume-based analyses) were entered as covariates. Given the non-significant differences in sensorimotor scores, we did not enter other covariates. Separate *t*-contrasts were run to test both volume and thickness increase and decrease, comparing NP vs. nNP, NP vs. noP, and nNP vs. noP. The same corrections for multiple comparisons as above were applied.

*Region-based morphometry.* In line with previous RBM analyses, different GLMs were run with the extracted GM volume and cortical thickness values as dependent variables, the pain sub-groups (NP, nNP, noP) as factor, and age, sex and TIV (the latter two variables only for the analysis of GM volumes) as covariates. As for previous RBM analyses, statistical significance was set at *p* ≤ 0.001 following Bonferroni’s method. All RBM analyses were performed using jamovi.

## 3 Results

ISNCSCI motor assessment was not available for one participant (see Supplementary Table S1). No significant between-group differences were observed in age (*U* = 834, *p* = 0.151) and sex distribution (*χ*^2^ = 3.03, *p* = 0.082). ISNCSCI scores of sensorimotor impairment were positively correlated with each other (motor and light touch: *ρ =* 0.484, *p* < 0.001; motor and pinprick: *ρ =* 0.446, *p* = 0.002; light touch and pinprick: *ρ =* 0.879, *p* < 0.001). No significant correlations emerged between TSI and the motor scores (*ρ =* −0.261, *p* = 0.087), or between TSI and the sensory scores (light touch: *ρ =* 0.139, *p* = 0.362; pinprick: *ρ =* 0.095, *p* = 0.536). No significant differences between the left and the right part of the body were found for the light touch examination (Mean ± Standard Deviation, SD; Left = 35.2 ± 10.9; Right = 34.9 ± 11.7; *W* = 0.867, *p* = 0.75), the pinprick examination (Left = 34 ± 12; Right = 33.6 ± 13.04; *W* = 0.255, *p* = 0.9), or the motor examination (Left = 35.5 ± 10.8; Right = 37.1 ± 10; *W* = 0.101, *p* = 0.06). Between-group comparisons based on the presence of pain, showed no significant difference in sensorimotor impairment (ISNCSCI scores; all *ps* > 0.650) or TSI (*p* = 0.092).

### 3.1 Differences between SCI and non-SCI

**Figure 1:**
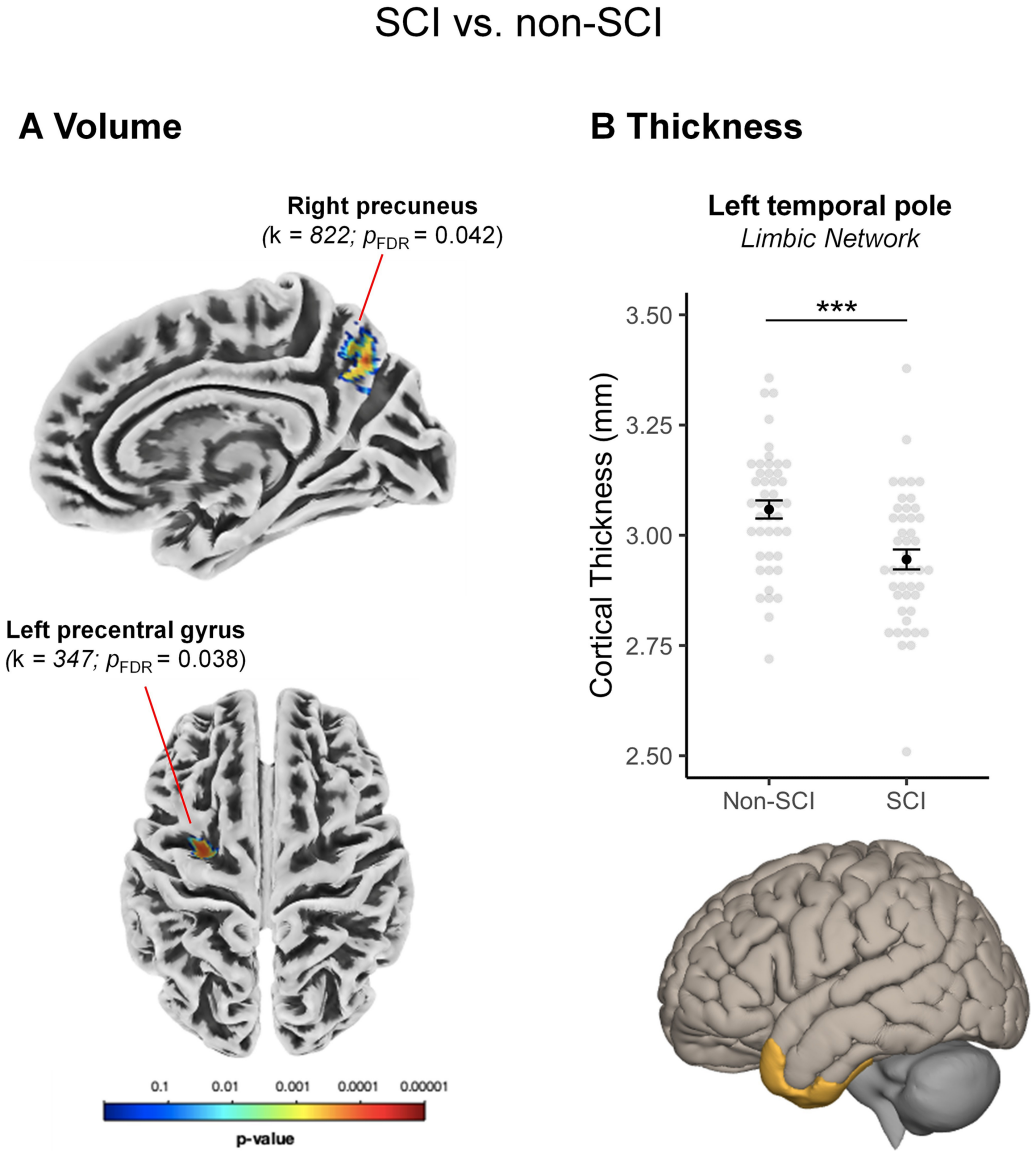
Between-group differences in gray matter volume and cortical thickness. Between-group differences in **(A)** gray matter volume and **(B)** cortical thickness. **(A)** Compared to the group without spinal cord injury (non-SCI), the SCI group showed smaller volume in the left precentral gyrus and a volume increase in the right precuneus, as indicated by the whole-brain analyses. **(B)** The region-based analyses within the Schaefer’s atlas indicated a reduction of cortical thickness in the left temporal pole. Mean ± standard error of the mean (in black) and individual raw data (light gray) are shown k: cluster size. ***p_Bonferroni_ ≤ 0.001.

*GM volume.* The whole-brain analysis revealed a significant volume reduction in the SCI group in the left sensorimotor network, namely, the left precentral gyrus (*p_FDR_* = 0.038), and a volume increase in a portion of the right precuneus within the ECN (*p_FDR_* = 0.042; see Table 2 and Figure 1A). The RBM analysis within the Schaefer’s atlas revealed no further significant between-group differences.

**Table 2:** Whole-brain analyses.

Main predictor	GM measure	Area	Cluster (voxels)	*x*	*y*	*z*	*p*_FDR_
SCI vs. non-SCI	Volume	Left PrG	347	−30	−6	50	*p* = 0.038
		Right PCu	822	8	−68	42	*p* = 0.042
ISNCSCI Motor	Volume	Right PrG	757	3	−22	80	*p* = 0.023
		Left PrG	1,608	−14	−28	54	*p* = 0.002
		Right TP	803	36	20	−26	*p* = 0.023
ISNCSCI Pinprick	Volume	Right OFC	674	34	26	−28	*p* = 0.033
		Left OFC	547	−34	27	20	*p* = 0.033

Main peaks of significant clusters. GM, grey matter; ISNCSCI, International Standard for Neurological Classification of Spinal Cord Injury; non-SCI, participants without spinal cord injury; OFC, orbitofrontal cortex; PCu, precuneus; PoG, postcentral gyrus; PrG, precentral gyrus; SCI, participants with spinal cord injury; TP, temporal pole.

*Cortical thickness.* Whole-brain analyses showed no significant between-group differences in cortical thickness, whereas the RBM analysis indicated reduced cortical thickness in the SCI group, in a portion of the left temporal pole within the limbic network (LimbicA_TempPole_1; *F_1, 87_ = 12.3*, *p_Bonferroni_* < 0.001, *η^2^_p_* = 0.124; Figure 1B).

### 3.2 Associations between GM morphometry and clinical variables

#### 3.2.1 Associations with TSI

**Figure 2:**
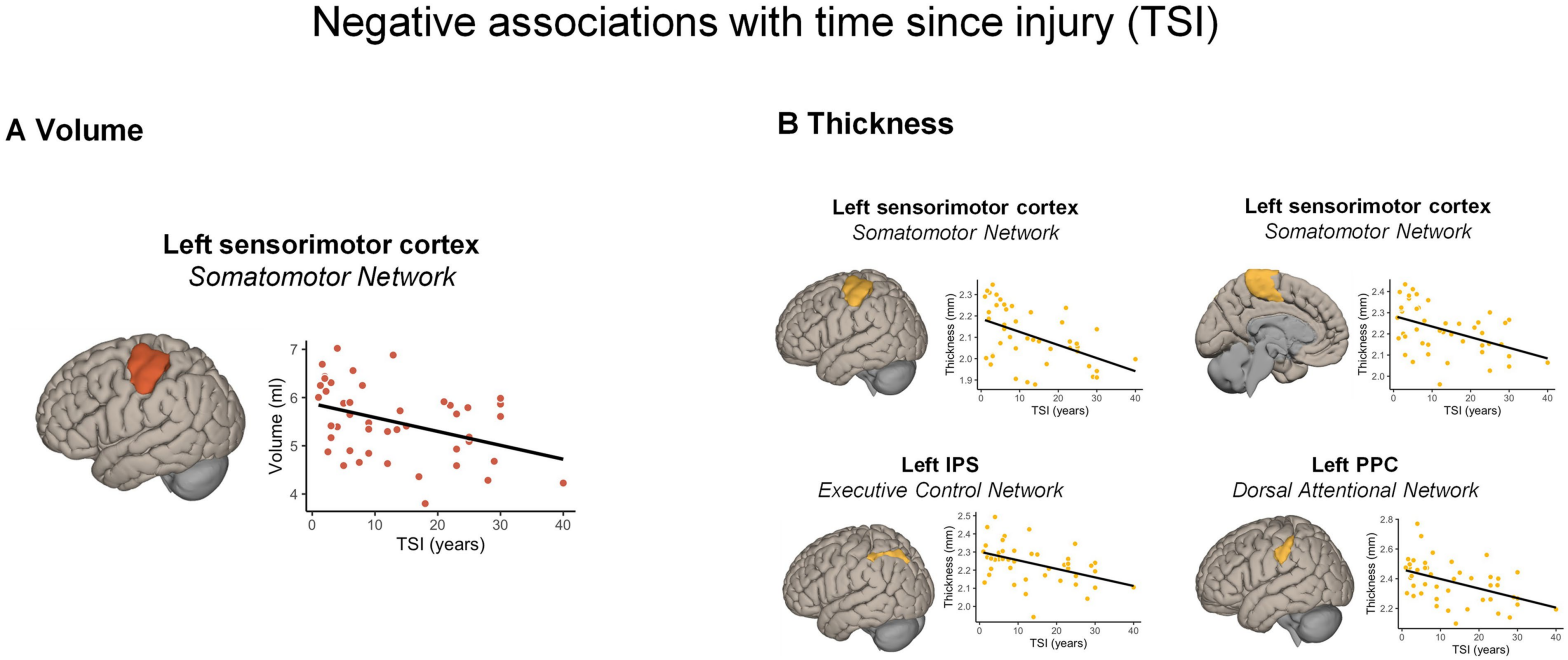
Significant morphometric changes negatively associated with time since injury. A longer TSI was associated with **(A)** a greater volume reduction and **(B)** cortical thickness reduction across different cortical regions, according to the Schaefer’s atlas. For visualization purposes, the scatterplots display correlations between raw values. IPS, intra-parietal sulcus; PPC, posterior parietal cortex; TSI, time since injury.

*GM volume.* The whole-brain analysis revealed no significant associations between GM volume and TSI. The RBM analysis (see Figure 2A; Supplementary Table S2) showed a negative association with TSI (i.e., the longer the TSI, the smaller the volume) in the left lateral sensorimotor areas (*β* = −0.33, *p_Bonferroni_* = 0.001).

*Cortical thickness.* The whole-brain analysis showed no significant positive or negative associations between cortical thickness and the TSI. Nonetheless, the RBM analysis indicated negative associations with TSI (i.e., the longer the TSI, the thinner the cortical thickness) with the left lateral (*ρ =* −0.471, *p_Bonferroni_* < 0.001) and dorso-medial sensorimotor areas (*ρ =* −0.430, *p_Bonferroni_* = 0.001), as well as with a part of the left posterior parietal cortex (PPC) in the DAN (*ρ =* −0.452, *p_Bonferroni_* = 0.001). Finally, a significant negative correlation emerged in a portion of the left intra-parietal sulcus (IPS; *ρ =* −0.450, *p_Bonferroni_* = 0.001) part of the ECN (Figure 2B).

#### 3.2.2 Associations with motor impairment—ISNCSCI motor scores

**Figure 3:**
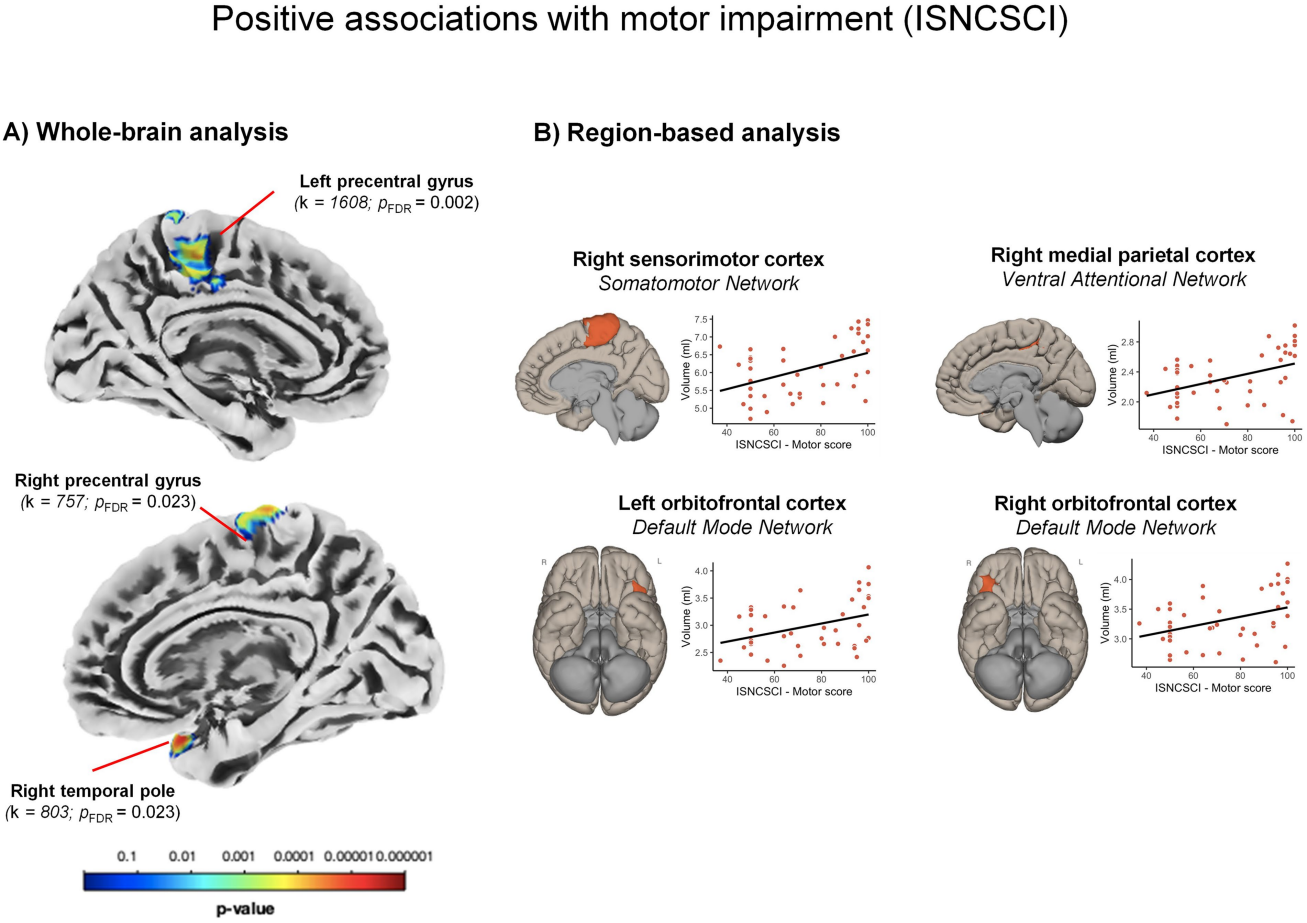
Significant volume changes positively associated with motor impairment. Significant positive associations between brain volume and the motor scores of the International Standard for Neurological Classification of Spinal Cord Injury (ISNCSCI) indicating greater volume reductions in participants with more severe motor impairment. **(A)** Results from whole-brain analyses. **(B)** Results from region-based analyses (Spearman correlations) within the Schaefer’s atlas. For visualization purposes, the scatterplots display correlations between raw values. k: cluster size.

*GM volume.* The whole-brain analysis revealed significant positive associations (i.e., the lower the motor scores, the smaller the volume) with clusters in the bilateral sensorimotor networks, especially the precentral gyri (both *ps_FDR_* ≤ 0.023; see Table 2 and Figure 3A), and in the right temporal pole (*p_FDR_* = 0.023), part of the limbic network.

The RBM analysis (see Figure 3B) further indicated the positive association between the motor scores and the volume within the sensorimotor network, specifically the dorsal-medial part of the right sensorimotor cortex (*β* = 0.41, *p_Bonferroni_* < 0.001; see Supplementary Table S2). Moreover, a positive association was observed in areas of the DMN, specifically in the bilateral orbitofrontal cortex (both *β* > 0.32, *p_Bonferroni_ ≤* 0.001). A positive correlation also emerged within the VAN in the left medial parietal cortex (*β* = 0.37, *p_Bonferroni_* < 0.001). No significant negative associations reached the significance level.

*Cortical thickness.* No negative or positive associations emerged between cortical thickness and the motor scores, neither at whole-brain nor at region-based level.

#### 3.2.3 Associations with sensory impairments—ISNCSCI light touch and pinprick scores

**Figure 4:**
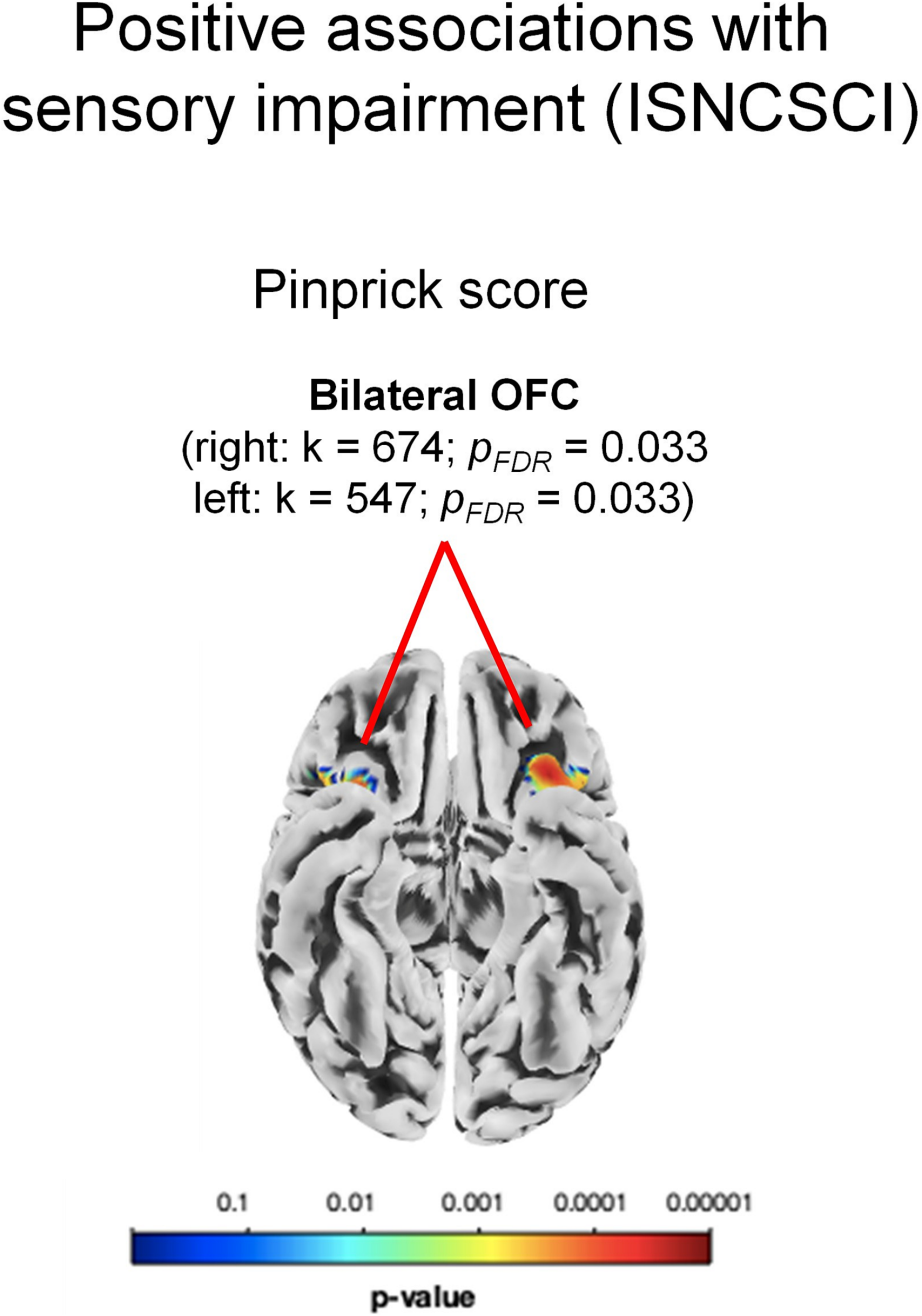
Significant morphometric changes positively associated with sensory impairment. Significant positive associations between brain volume and the pinprick score of the International Standard for Neurological Classification of Spinal Cord Injury (ISNCSCI) indicating greater volume reduction in the bilateral orbitofrontal cortex (OFC) in participants with more severe sensory impairments. k: cluster size.

*GM volume.* The whole-brain and the RBM analyses showed no significant associations with the light touch score. The pinprick scores were positively associated with clusters in the bilateral posterior OFC (both *ps_FDR_* = 0.033) as revealed by the whole-brain analysis (see Table 2; Figure 4). These correlations indicated that a lower sensory score was associated with smaller GM volume in the aforementioned areas. No significant negative associations were detected.

*Cortical thickness.* No significant associations were observed between cortical thickness and the sensory scores, neither at whole-brain nor at region-based level.

#### 3.2.4 Interactions between TSI and motor deficits

The GLMs did not reveal any significant interaction between TSI and the motor scores in predicting changes of GM volume (all *Fs* ≤1.37; all *ps* ≥ 0.25) or cortical thickness (all *Fs* ≤ 1.37; all *ps* ≥ 0.25). See also Supplementary Table S3.

### 3.3 The effect of pain on GM morphometry

*GM volume.* The whole-brain analyses (see Supplementary Table S4 and Supplementary Figure S1, in the Supplementary Material) revealed a significant volume reduction in SCI individual with NP, compared to noP, in different brain clusters including the right precentral gyrus (*p_FDR_* = 0.03), the right postcentral gyrus/parietal operculum (*p_FDR_* < 0.001), right putamen (*p_FDR_* = 0.004), as well as the left (ventral dentate nucleus and crus 2; *ps_FDR_* ≤ 0.003) and the right cerebellum (lobule 7b; *p_FDR_* = 0.001). Similarly, a volume reduction was observed in individuals with nNP (vs. noP) in the right postcentral gyrus (*p_FDR_* = 0.013), the right putamen (*p_FDR_* = 0.013), and the cerebellar Crus 1 and 2 (*p_FDR_* ≤ 0.020). No differences were observed between NP and nNP. Likewise, no significant volume increase emerged in any comparison. Finally, the RBM analyses revealed no significant between-group differences.

*Cortical thickness.* Whole-brain analyses and RBM analyses revealed no significant between-group differences in cortical thickness.

## 4 Discussion

Our study revealed distinct patterns of morphological brain reorganization in chronic SCI, characterized by motor cortex atrophy, precuneal expansion, and temporal pole thinning (SCI vs. non-SCI comparisons), as well as GM volume and thickness reductions associated with TSI and the severity of sensorimotor impairment. These changes were observed not only in the well-known cortical sensorimotor areas, but also in frontal, temporal and parietal nodes of functional networks, including the default mode, attentional and executive control networks. An explorative analysis on morphometric changes associated with chronic pain, showed extended volume reductions in the bilateral sensorimotor cortexes, basal ganglia and cerebellum, in both SCI individuals with NP and nNP. These results extend current knowledge on brain reorganization after spinal cord injury by specifically focusing on individuals with chronic SCI—a stage in which secondary health conditions and long-term neuroplastic changes are particularly relevant—and by adopting a functionally informed, network-based perspective to better integrate structural, functional, and behavioral alterations observed in this population.

### 4.1 Morphometric changes within the sensorimotor network

Consistent with previous literature (4, 6, 14, 16), our findings indicate that SCI leads to degeneration within the central sensorimotor networks of the CNS, likely reflecting progressive deterioration of efferent motor pathways and afferent sensory pathways (12). This neurodegeneration may be supported by processes such as myelin degradation and increased iron accumulation (8), contributing to structural decline in primary somatosensory cortices and subcortical structures. These structural alterations, as well as the broader network-level changes discussed below (see section 4.2), were reflected by both cortical thickness [indicating changes in cortical laminar architecture (34)] and GM volume (which integrates cortical thickness, surface area, and cortical folding), highlighting the importance of considering both metrics in a complementary fashion, as suggested by Hutton and colleagues (45) in their investigation of brain aging.

Our results, based on a sample with a wide range of TSI (1–40 years) adds complementary evidence to prior longitudinal studies (4, 6) showing that GM degeneration within the sensorimotor network can continue for several years following SCI. Nonetheless, the trajectory of brain degeneration is also known to be modulated by individual clinical profiles and, specifically, by the extent of neurological and functional recovery (5, 6, 10), with “non-recoverers” showing steeper trajectories of spinal cord volume loss and iron accumulation in the sensorimotor cortexes. In this context, the secondary analyses indicated that cortical volume was associated with the severity of motor symptoms, with lower GM volume in those individuals with more severe motor impairments. The effects of motor deprivation seemed particularly relevant in the dorso-medial part of sensorimotor cortexes (especially the precentral gyrus, see the whole-brain analysis), i.e., those areas processing sensorimotor activity of the lower limbs. In our study, however, we did not observe any significant interaction between TSI and the severity of motor impairment in predicting morphometric changes, either within or beyond sensorimotor networks. One possible explanation is that the cross-sectional design limits the ability to capture such interactive effects. Indeed, some participants with long-standing injuries exhibited only mild neurological deficits, while others with relatively short TSI showed severe sensorimotor impairments, potentially masking cumulative or synergistic effects of these variables. Longitudinal studies starting from the acute/subacute phase post-SCI are better suited to more accurately characterize the trajectory of structural brain alterations and to disentangle the combined influences of injury duration and clinical severity.

Although exploratory in nature, our data suggest that the presence of pain is associated with volume reduction in a network of regions including the sensorimotor cortices, basal ganglia, and cerebellum. This pattern of atrophy is consistent with the so-called pain matrix—a set of interacting networks involved in modulating both the sensory and cognitive-emotional dimensions of pain (46). Interestingly, our results did not reveal morphometric patterns specifically associated with NP, but rather a general effect of both NP and nociceptive pain. In addition, no significant differences emerged when directly comparing individuals with NP to those with nNP. NP is thought to result from maladaptive plasticity occurring throughout the neuraxis above the lesion level (19, 21, 47), including spinal cord atrophy and bidirectional morphometric changes in brain regions involved in pain processing—such as the insula, thalamus, cingulate cortex, and primary sensorimotor areas—potentially leading to dysregulation of ascending and descending brain–spinal pathways (21, 47). In our study, when each pain group (NP and nNP) was compared to pain-free individuals, the most notable differences were observed in the bilateral somatosensory cortexes, the basal ganglia and the cerebellum, with regions showing smaller volume in both pain groups. Changes in the motor cortex associated with pain have already been reported (21), however the directionality of these relationships is yet to be established, and alterations in M1 in chronic pain are still object of debate (48). Our results may indicate that chronic atrophy of the sensorimotor cortex may be linked to more severe pain, irrespectively of its etiology. Future studies with a larger sample sizes and an in-depth characterization of pain may better elucidate the brain mechanisms of pain in chronic SCI, a topic of high clinical relevance given the recent advancements in non-invasive pain modulation (49), which may open to target interventions for pain management in SCI.

### 4.2 Morphometric changes beyond the sensorimotor network

Compared to non-SCI, the SCI group showed a significant increase in GM volume in the right precuneus—specifically within the ECN – and reduced cortical thickness in the left temporal pole within the limbic network.

Our results on the right precuneus confirm the preliminary findings in a smaller sample of chronic complete thoracic SCI participants by Murayama and colleagues (20). The precuneus has been implicated in various higher-order cognitive functions, including visuo-spatial imagery, self-referential processing, and aspects of consciousness and attention (50, 51). Interestingly, a functional MRI (fMRI) study by Wenderoth and colleagues (52) on bimanual coordination, showed that complex bimanual coordination requires an increased activation of the precuneus, highlighting its role in shifting attention between different locations in space, to monitor bimanual trajectories, and thus coordinating complex movements. Thus, as interface between higher-executive functions and sensorimotor integration (53, 54), a precuneal expansion may reflect an adaptive response to altered sensorimotor input (especially from the lower limbs), supporting the implementation and consolidation of new coordinated motor schemes of the upper body, supported by structural connections between the precuneus, premotor, and supplementary motor areas (53).

In contrast, the SCI group exhibited reduced cortical thickness in the left temporal pole within the limbic network. Degeneration of the temporal pole has previously been reported by Chen et al. (18), who observed reduced GM volume in the right temporal pole using a whole-brain approach in a smaller, mixed cohort of subacute and chronic SCI participants. These findings suggest that structural alterations in this region may emerge early after injury and persist into the chronic stage, potentially reflecting long-term involvement of limbic network in SCI-related neurodegeneration. Notably, the temporal pole is known to play a role in social and emotional processing, memory, and language (55). Reductions in cortical thickness in this region might be associated with secondary health conditions reported in SCI, such as chronic stress and emotional dysregulation (56). In fact, the areas of limbic network, involving the temporal pole and part of the OFC (39), are structurally connected with cortical and subcortical areas, e.g., the cingulate cortex, the thalamus and the amygdala (57), that play an important role in pain processing and regulation. Not surprisingly, these areas, as well as the sensorimotor cortexes, undergo morphological and functional changes due to NP in SCI (21, 24, 26). Importantly, a recent study in chronic SCI showed that surgical treatment of dorsal roots not only induced a significant analgesic effect, but also normalized patterns of functional connectivity within the limbic network and between limbic network and primary sensorimotor areas (58), thus making this network particularly relevant for SCI individuals suffering from chronic pain. However, our exploratory analysis of pain did not reveal major morphometric alterations within limbic regions typically associated with NP. Future studies with a more balanced sample size and a comprehensive pain assessment are needed to further elucidate the relationship between structural alterations in the limbic network, pain processing, and associated emotional distress.

We further explored the relationship between GM structure and clinical variables, including TSI and the degree of sensorimotor impairment (secondary outcomes), and found them associated with volume and thickness changes in fronto-temporo-parietal regions within different functional networks. First, we observed a correlation between TSI and GM reductions in the IPS and the PPC—components of the ECN and DAN. It is important to note that both areas of the parietal cortex are involved in voluntary attentional control (59, 60), in particular dorsal attentional activity, mediated by a network of fronto-parietal areas, sustains top-down attentional abilities, driving our attention to the surrounding space in a goal-oriented manner. A degeneration of these networks might potentially contribute to cognitive deficits observed in SCI, with a progressive deterioration over time (32, 61). Importantly, a recent meta-analysis comparing cognitive performance between individuals with and without SCI (62), found that the SCI group experienced reduced cognitive abilities, particularly in attentional tasks and executive functioning, in line with the morphological changes observed in our study. Our results extend the results by Chen and colleagues (18), who observed structural and functional alterations in cognition-related nodes of the ECN, also demonstrating an association between fronto-cingulate and fronto-precuneal functional connectivity with motor impairments. Moreover, we observed an association of motor impairments and volume decrease with the medial parietal cortex within the VAN. This network is involved in selecting the most salient information from the body and the external environment, playing a pivotal role in guiding attention and behavior (17). These findings support the idea that SCI affects not only the sensorimotor system, but also higher-order attentional networks, mediating top-down and bottom-up processes integrating sensorimotor information with higher cognitive functioning. However, the connection between SCI, cognitive impairment, and structural-functional brain changes remains unexplored. As our study lacked neuropsychological data, future research incorporating detailed cognitive assessments is essential to clarify these associations.

Lastly, we observed consistent associations between sensory and motor scores and GM volume loss in frontal nodes of the DMN, namely the bilateral posterior OFC. While traditionally linked to internally oriented processes (e.g., self-referential thought, mind-wandering) (63), the DMN is being increasingly studied due to its involvements in higher cognitive tasks (64), and chronic pain (65), showing dynamic interactions with sensorimotor and attentional networks. Studies in chronic SCI have reported mixed patterns of hypoconnectivity or hyperconnectivity between the sensorimotor regions (known to undergo structural changes following SCI) and the DMN (28), as well as between DMN components (e.g., the superior frontal gyrus) (66) and nodes of the VAN (i.e., anterior cingulate and supplementary motor areas) (28). Moreover, our findings are consistent with previous morphometric studies conducted on smaller and more heterogeneous SCI samples (17, 18) which reported degeneration of bilateral OFC—a region involved in emotional and behavioral regulation, including the processing of pain-related emotional valence (67)—and found that OFC atrophy correlated with the severity of sensorimotor impairment. As discussed above, the OFC is structurally connected with limbic structures, insula, the cingulate cortex [both involved in pain processing, and, importantly, the somatosensory cortex (68)]. Taken together, our findings suggest that disruption of sensorimotor pathways following SCI may lead to secondary degeneration in structurally and functionally connected regions, including those involved in pain processing and emotional regulation, potentially contributing to the development of affective disorders and chronic neuropathic pain in this population. Moreover, the involvement of DMN and attentional network nodes in relation to clinical severity highlights the need to conceptualize SCI as a condition that affects widespread brain systems, beyond the classic sensorimotor circuits.

This study has some limitations that should be considered. First, a comprehensive assessment of cognitive deficits, affective symptoms, and pain—as well as their longitudinal trajectories—is essential to clarify the causal relationship between structural brain changes in functional networks and the development of these clinically relevant outcomes. However, such data were not available for our sample, except for pain, whose associated brain changes were investigated at an exploratory level. Future research may directly investigate the effects of such secondary health changes on brain morphology. In addition, the cross-sectional nature of this investigation limits causal inferences regarding the evolution of morphometric changes. Longitudinal studies are needed to determine the trajectory of structural alterations beyond the sensory-motor networks. Moreover, our study examined brain changes from a broad perspective, combining a conventional whole-brain approach with a network-based perspective spanning the entire cortex. This strategy necessarily involved a large number of comparisons and stringent correction for multiple testing, which tend to increase the sensitivity to larger effects, possibly masking subtler or more localized changes. On the other hand, although corrections were applied within each analysis, no dataset-wide multiplicity correction was implemented. Consequently, exploratory findings from secondary analyses should be regarded as hypothesis-generating and interpreted with caution. Future studies may benefit from more specific hypotheses about the relationship between secondary health conditions and brain reorganization, and from focused analyses on targeted brain networks. Such an approach could further enhance the sensitivity, the robustness, and the interpretability of the findings.

We also acknowledge that the sample is clinically heterogeneous in terms of neurological level of injury and lesion completeness. Individual injury characteristics are likely to influence brain reorganization [e.g., lesion completeness (18)], and are associated with secondary health complications—including autonomic dysregulation and related conditions—that warrant further investigation. Although the impact of these factors was not directly tested due to the sample characteristics (e.g., the imbalance between complete and incomplete lesions; see Table 1), we believe that ISNCSCI sensorimotor scores can account for some of this clinical variability. As such, the ISNCSCI sensorimotor scores represent the main clinical variable of interest in numerous neuroimaging studies in SCI, also with heterogeneous samples (6, 10). Finally, future works could integrate multimodal imaging, also assessing white matter structural integrity and functional connectivity of the brain areas highlighted from this study.

In conclusion, the present study advances our understanding of brain reorganization in chronic SCI, a stage marked by long-term neuroplastic and secondary changes, through a functionally informed, network-based approach aimed at integrating structural, functional, and behavioral alterations. Our results are indicative of widespread structural reorganization both within and beyond the sensorimotor network. These alterations involve key regions implicated in emotion regulation, pain processing, and cognitive functioning, and were associated with both the duration and severity of injury. Together, these findings highlight that the neurobiological consequences of SCI extend far beyond sensorimotor pathways and may contribute to the cognitive and emotional sequelae frequently observed in this population. Future longitudinal studies are warranted to elucidate the causal mechanisms underlying these changes and to assess their potential as imaging biomarkers of secondary health conditions in chronic SCI.

## Data Availability

The data that support the findings of this study are available from the corresponding author upon reasonable request. Requests to access these datasets should be directed to giuseppe.zito@paraplegie.ch.
