# Supplementary material for: Chronic spinal cord injury is associated with morphometric brain changes in functional networks beyond the sensorimotor system
Source: Front Neurol. 2025 Dec 8;16:1672506. doi: 10.3389/fneur.2025.1672506 (PMC12722829; doi:10.3389/fneur.2025.1672506)
Supplement: Supplementary file 1 [file Data_Sheet_1.docx]

Supplementary Material

**Table S1. Individual demographic and clinical data of the SCI sample**

| **ID** | **Sex** | **Age (years)** | **TSI (years)** | **SCI**  **Etiology** | **AIS Grade** | **Neurological level^a^** | **ISNCSCI**  **Motor L/R** | **ISNCSCI**  **Motor Total** | **ISNCSCI**  **Light touch L/R** | **ISNCSCI**  **Light touch Total** | **ISNCSCI**  **Pinprick L/R** | **ISNCSCI**  **Pinprick Total** | **Pain** |
| --- | --- | --- | --- | --- | --- | --- | --- | --- | --- | --- | --- | --- | --- |
| 01 | M | 59 | 9 | Traumatic - Road accident | A | T3/T3 | 50 / 50 | 100 | 21 / 21 | 42 | 21 / 21 | 42 | nNP |
| 02 | F | 58 | 7.5 | Traumatic - Post-surgery | D | T1/T1 | 50 / 49 | 99 | 38 / 49 | 87 | 38 / 49 | 87 | NP |
| 03 | M | 52 | 25 | Traumatic - Road accident | B | T6/T6 | 25 / 25 | 50 | 27 / 27 | 54 | 27 / 27 | 54 | nNP |
| 04 | M | 39 | 12.9 | Traumatic - Road accident | B | T1/T1 | 20 / 25 | 45 | 22 / 22 | 44 | 21 / 22 | 43 | noP |
| 05 | M | 51 | 13.5 | Traumatic - Road accident | D | T2/T2 | 49 / 47 | 96 | 49 / 55 | 104 | 42 / 47 | 89 | noP |
| 06 | M | 34 | 6.5 | Traumatic - Road accident | A | T3/T3 | 25 / 25 | 50 | 21 / 21 | 42 | 21 / 20 | 41 | noP |
| 07 | M | 51 | 23 | Traumatic - Fall | A | T9/T9 | 33 / 31 | 64 | 38 / 37 | 75 | 38 / 38 | 76 | noP |
| 08 | M | 35 | 2.2 | Non-traumatic - Tumor | D | T2/T2 | 50 / 50 | 100 | 31 / 37 | 68 | 31 / 37 | 68 | NP |
| 09 | F | 35 | 5 | Traumatic - Fall | D | T11/T11 | 30 / 41 | 71 | 43 / 46 | 89 | 46 / 46 | 92 | NP |
| 10 | M | 20 | 3 | Traumatic - Post-surgery | D | T3/T3 | 47 / 49 | 96 | 38 / 39 | 77 | 28 / 23 | 51 | noP |
| 11 | M | 58 | 14 | Traumatic - Road accident | C | T2/T2 | 29 / 35 | 64 | 35 / 37 | 72 | 21 / 19 | 40 | NP |
| 12 | F | 19 | 1.6 | Traumatic - Fall | D | S1/L3 | 50 / 50 | 100 | 53 / 53 | 106 | 56 / 56 | 112 | nNP |
| 13 | M | 36 | 8 | Traumatic - Fall | D | L5/L1 | 48 / 48 | 96 | 48 / 48 | 96 | 40 / 48 | 88 | NP |
| 14 | M | 45 | 23 | Traumatic - Not specified | D | C7/C7 | 24 / 46 | 70 | 34 / 12 | 46 | 34 / 12 | 46 | nNP |
| 15 | F | 41 | 28 | Traumatic - Road accident | C | C6/C5 | 35 / 22 | 57 | 34 / 34 | 68 | 34 / 35 | 69 | nNP |
| 16 | M | 39 | 22 | Traumatic - Road accident | A | T10/T10 | 25 / 25 | 50 | 37 / 36 | 73 | 36 / 34 | 70 | nNP |
| 17 | M | 41 | 1.3 | Traumatic - Head-first dive | D | C5/C1 | 45 / 41 | 86 | 27 / 26 | 53 | 4 / 6 | 10 | nNP |
| 18 | F | 53 | 2.5 | Traumatic - Road accident | B | L1/L3 | 34 / 34 | 68 | 50 / 50 | 100 | 48 / 47 | 95 | noP |
| 19 | M | 51 | 25 | Traumatic - Road accident | C | T12/T12 | 35 / 29 | 64 | 54 / 49 | 103 | 47 / 51 | 98 | noP |
| 20 | M | 48 | 24.8 | Traumatic - Road accident | A | T8/T8 | 25 / 25 | 50 | 30 / 30 | 60 | 30 / 30 | 60 | noP |
| 21 | F | 34 | 9 | Traumatic - Fall | B | L2/L1 | 34 / 33 | 67 | 43 / 43 | 86 | 43 / 43 | 86 | nNP |
| 22 | M | 56 | 3 | Traumatic - Contusion | D | C1/C1 | 49 / 45 | 94 | 28 / 32 | 60 | 27 / 18 | 45 | NP |
| 23 | M | 50 | 30 | Traumatic - Fall | A | S1/S1 | 50 / 50 | 100 | 52 / 52 | 104 | 52 / 52 | 104 | nNP |
| 24 | M | 50 | 4 | Traumatic - Road accident | D | C3/C3 | 43 / 50 | 93 | 32 / 33 | 65 | 32 / 26 | 58 | NP |
| 25 | M | 32 | 9 | Traumatic - Head-first dive | D | C2/C2 | 42 / 47 | 89 | 41 / 32 | 73 | 37 / 33 | 70 | noP |
| 26 | F | 32 | 12 | Traumatic - Road accident | A | T6/T6 | 25 / 25 | 50 | 28 / 28 | 56 | 27 / 27 | 54 | nNP |
| 27 | F | 54 | 23 | Traumatic - Road accident | A | T12/T12 | 25 / 25 | 50 | 40 / 40 | 80 | 40 / 40 | 80 | nNP |
| 28 | M | 58 | 6 | Traumatic - Road accident | A | T3/T3 | 25 / 25 | 50 | 21 / 21 | 42 | 20 / 20 | 40 | nNP |
| 29 | M | 59 | 5 | Traumatic - Ski accident | D | C2/C2 | 41 / 40 | 81 | 29 / 29 | 58 | 31 / 29 | 60 | nNP |
| 30 | M | 37 | 30 | Traumatic - Not specified accident | D | L4/L3 | 48 / 50 | 98 | 50 / 52 | 102 | 45 / 47 | 92 | noP |
| 31 | M | 55 | 29 | Traumatic - Road accident | D | C8/C4 | 45 / 49 | 94 | 54 / 56 | 110 | 54 / 56 | 110 | nNP |
| 32 | F | 57 | 40 | Traumatic - Ski accident | D | T1/T1 | 39 / 48 | 87 | 39 / 36 | 75 | 46 / 40 | 86 | NP |
| 33 | M | 43 | 21 | Traumatic - Contusion | A | C7/C6 | 14 / 23 | 37 | 14 / 16 | 30 | 11 / 11 | 22 | noP |
| 34 | M | 25 | 6 | Traumatic - Road accident | D | C2/C2 | 39 / 42 | 81 | 40 / 33 | 73 | 39 / 34 | 73 | NP |
| 35 | M | 34 | 15 | Traumatic - Road accident | A | T10/T10 | 25 / 25 | 50 | 36 / 38 | 74 | 34 / 36 | 70 | nNP |
| 36 | F | 46 | 4 | Traumatic - Fall | A | T10/T10 | 25 / 25 | 50 | 35 / 35 | 70 | 36 / 36 | 72 | NP |
| 37 | M | 49 | 2 | Traumatic - Surf accident | A | C6/C4 | n.a | n.a. | 11 / 8 | 19 | 9 / 8 | 17 | NP |
| 38 | M | 38 | 2 | Traumatic - Not specified | D | C3/C3 | 50 / 50 | 100 | 31 / 31 | 62 | 46 / 35 | 81 | nNP |
| 39 | F | 47 | 6 | Traumatic - Road accident | C | T4/T4 | 32 / 39 | 71 | 47 / 48 | 95 | 46 / 48 | 94 | nNP |
| 40 | M | 24 | 1 | Traumatic - Road accident | A | T11/T11 | 25 / 25 | 50 | 37 / 35 | 72 | 37 / 35 | 74 | NP |
| 41 | M | 55 | 12 | Traumatic - Road accident | D | C8/C5 | 50 / 45 | 95 | 42 / 34 | 76 | 38 / 39 | 77 | nNP |
| 42 | M | 57 | 17 | Traumatic - Not specified accident | A | T1/T1 | 25 / 25 | 50 | 20 / 20 | 40 | 17 / 17 | 34 | NP |
| 43 | F | 37 | 18 | Traumatic - Road accident | A | C3/C3 | 23 / 24 | 47 | 19 / 22 | 41 | 20 / 22 | 42 | NP |
| 44 | M | 47 | 30 | Traumatic - Road accident | C | T3/T3 | 26 / 30 | 56 | 33 / 35 | 68 | 33 / 35 | 68 | nNP |
| 45 | F | 55 | 3 | Traumatic - Fall | D | C5/C6 | 34 / 46 | 80 | 33 / 34 | 67 | 47 / 56 | 103 | NP |

*Note*: ^a^motor and sensory levels are reported. AIS: American Spinal Injury Association Impairment Scale; ISNCSCI: International Standards for Neurological Classification of Spinal Cord Injury; L/R: left and right sides of the body; n.a.: not available; NP: neuropathic pain; nNP: non-neuropathic pain; noP: no pain; TSI: Time Since Injury.

**Table S2. Region-based morphometry**

| **Main predictor** | **GM Measure** | **Schaefer's atlas label** | **Region** | **Statistics** | ***p*_Bonferroni_** |
| --- | --- | --- | --- | --- | --- |
| TSI | Volume | SomMotA_1 | Left lateral SM areas | F_1,40_ = 11.03, *η²_p_* = 0.216,  *β* = -0.33 | *p* = 0.001 |
| TSI | Thickness | SomMotA_1 | Left lateral SM areas | *ρ* = -0.471 | *p* < 0.001 |
| TSI | Thickness | SomMotA_2 | Left dorso-medial SM areas | *ρ* = -0.430 | *p* = 0.001 |
| TSI | Thickness | DorsAttnB_PostC_1 | Left PPC | *ρ* = -0.452 | *p* = 0.001 |
| TSI | Thickness | ContA_IPS_1 | Left IPS | *ρ* = -0.450 | *p* = 0.001 |
| ISNCSCI Motor | Volume | SomMotA_4 | Right dorso-medial SM areas | F_1,39_ = 15.9,  *η²_p_* = 0.29,  *β* = 0.41 | *p* < 0.001 |
| ISNCSCI Motor | Volume | DefaultB_PFCv_1 | Right OFC | F_1,39_ = 10.81,  *η²_p_* = 0.22,  *β* = 0.33 | *p* = 0.001 |
| ISNCSCI Motor | Volume | DefaultB_PFCv_1 | Left OFC | F_1,39_ = 11.73,  *η²_p_* = 0.23,  *β* = 0.37 | *p* < 0.001 |
| ISNCSCI Motor | Volume | SalVentAttnA_PartMed_1 | Left medial parietal cortex | F_1,39_ = 11.67,  *η²_p_* = 0.23,  *β* = 0.37 | *p* < 0.001 |

*Note*: GM: grey matter; ISNCSCI: International Standards for Neurological Classification of Spinal Cord Injury; IPS: intra-parietal sulcus; OFC: orbito-frontal cortex; PPC: posterior parietal cortex; SM = sensorimotor; TSI: Time Since Injury.

**Table S3. Interactions between TSI and ISNCSCI motor scores**

| **GM Measure** | **Schaefer's atlas label** | **Region** | **Statistics - Interaction** |
| --- | --- | --- | --- |
| Volume | SomMotA_1 | Left lateral SM areas | *F*_1,37_ = 0.15; *p =* 0.697; *η²_p_* = 0.004 |
| Volume | SomMotA_4 | Right dorso-medial SM areas | *F*_1,37_ = 0.002; *p =* 0.965; *η²_p_* = 0 |
| Volume | DefaultB_PFCv_1 | Right OFC | *F*_1,37_ = 0.64; *p =* 0.43; *η²_p_* = 0.017 |
| Volume | DefaultB_PFCv_1 | Left OFC | *F*_1,37_ = 0.105; *p =* 0.747; *η²_p_* = 0.003 |
| Volume | SalVentAttnA_PartMed_1 | Left medial parietal cortex | *F*_1,37_ = 1.37; *p =* 0.25; *η²_p_* = 0.036 |
| Thickness | SomMotA_1 | Left lateral SM areas | *F*_1,39_ = 0.001; *p =* 0.988; *η²_p_* = 0 |
| Thickness | SomMotA_2 | Left dorso-medial SM areas | *F*_1,39_ = 1.3; *p =* 0.261; *η²_p_* = 0.032 |
| Thickness | DorsAttnB_PostC_1 | Left PPC | *F*_1,39_ = 0.29; *p =* 0.6; *η²_p_* = 0.007 |
| Thickness | ContA_IPS_1 | Left IPS | *F*_1,39_ = 0.02; *p =* 0.885; *η²_p_* = 0.001 |

*Note*: grey matter; ISNCSCI: International Standards for Neurological Classification of Spinal Cord Injury; IPS: intra-parietal sulcus; non-SCI: individuals without spinal cord injury; OFC: orbitofrontal cortex; PPC: posterior parietal cortex; SCI: individuals with spinal cord injury; SM = sensorimotor; TSI: Time Since Injury.

**Table S4. Whole-brain analyses - morphometric changes associated to pain**

| **Main predictor** | **GM Measure** | **Area** | **Cluster (voxels)** | **x** | **y** | **z** | ***p*_FDR_** |
| --- | --- | --- | --- | --- | --- | --- | --- |
| Pain (NP < noP) | Volume | Right PrG (medial) | 990 | 3 | 8 | 57 | *p* = 0.03 |
| Pain (NP < noP) | Volume | Right PoG/parietal operculum | 3584 | 60 | -10 | 21 | *p* < 0.001 |
| Pain (NP < noP) | Volume | Right Putamen | 1591 | 26 | 10 | -8 | *p* = 0.004 |
| Pain (NP < noP) | Volume | Left Cerebellum -VDN / left fusiform gyrus | 5656 | -24 | -62 | -16 | *p* <0.001 |
| Pain (NP < noP) | Volume | Left Cerebellum – Crus 2 | 2075 | -21 | -70 | -40 | *p =* 0.002 |
| Pain (NP < noP) | Volume | Right Cerebellum – Lobule 7b | 1911 | 36 | -64 | -51 | *p =* 0.001 |
| Pain (nNP < noP) | Volume | Right PoG/SMG | 1203 | 66 | -21 | 21 | *p =* 0.013 |
| Pain (nNP < noP) | Volume | Right Putamen | 1155 | 27 | 16 | 2 | *p =* 0.013 |
| Pain (nNP < noP) | Volume | Left Cerebellum – Crus 2 | 1337 | -26 | -75 | -39 | *p =* 0.020 |
| Pain (nNP < noP) | Volume | Right Cerebellum –Crus 1 | 1724 | 38 | -80 | -40 | *p =* 0.010 |

*Note*: main peaks of significant clusters. GM: grey matter; NP = SCI participants with neuropathic pain; nNP = SCI participants without neuropathic pain but with nociceptive pain; noP: SCI participants with no pain; PoG: postcentral gyrus; PrG: precentral gyrus; VDN: ventral dentate nucleus.


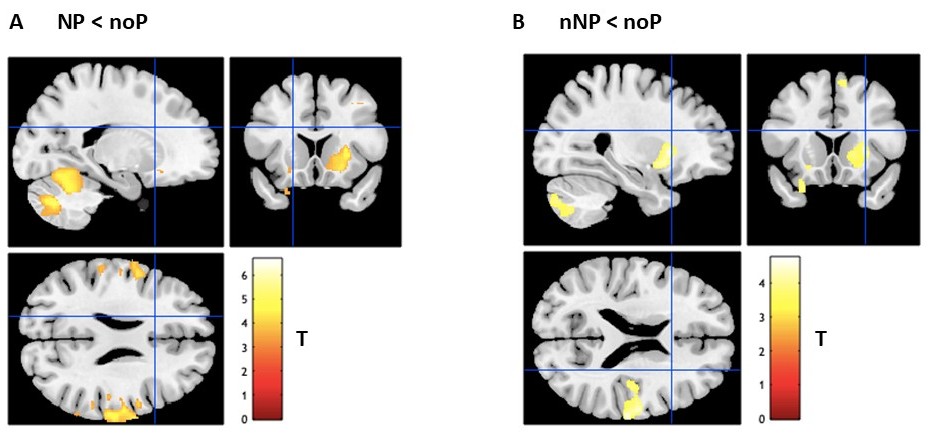


**Figure S1. Volume reduction associated to pain.** Significant clusters of reduced volume in A) individual with spinal cord injury (SCI) suffering from neuropathic pain (NP) compared to pain-free SCI (noP), and in B) SCI with nociceptive pain (non-neuropathic, nNP) compared to noP. Common patterns of reduced volume in sensorimotor cortexes, cerebellum, and basal ganglia were observed in both NP and nNP.
